# A case of septic arthritis of the temporomandibular joint with necrotic peri-articular infection and Lemierre’s syndrome: an unusual presentation

**DOI:** 10.1007/s10006-020-00921-z

**Published:** 2020-11-05

**Authors:** Mats Døving, Erik Egeland Christensen, Lars Peder Huse, Øystein Vengen

**Affiliations:** 1 Department of Maxillofacial Surgery, Oslo University Hospital Ullevål, PO Box 4956, Nydalen, 0424 Oslo, Norway; 2 Department of Infectious diseases, Oslo University Hospital Ullevål, Oslo, Norway; 3 Institute of Clinical Medicine, University of Oslo, Oslo, Norway; 4 Department of Cardiothoracic Surgery, Oslo University Hospital Ullevål, Oslo, Norway

**Keywords:** Temporomandibular joint, Septic arthritis, Sepsis, Lemierre syndrome, Brain abscess

## Abstract

**Background:**

Septic arthritis of the temporomandibular joint (TMJ) is rare. It usually causes isolated, locoregional symptoms related to the infected intra-articular space but may also cause fever and malaise.

**Case report:**

We present a case of a 72-year-old male with septic arthritis of the TMJ complicated by extensive peri-articular necrosis, septic shock, cerebral abscess, Lemierre’s syndrome, and a pathological fracture of the mandibular condyle.

**Conclusion:**

Case reports describing such a severe course of the disease are few. Moreover, this is the first report of septic arthritis of the TMJ to cause Lemierre’s syndrome.

## Introduction

Septic arthritis of the temporomandibular joint (TMJ) is a rare disease which usually presents with pain, pre-auricular swelling, lymphadenopathy, trismus, and malocclusion [1, 2]. In addition, fever and malaise may be present [3]. As a result of joint effusion, increased joint space can be seen on imaging studies such as orthopantomogram, computer tomography (CT), cone-beam CT (CBCT), and magnetic resonance imaging (MRI) [4].

Septic arthritis of the TMJ is caused by hematogenous spread, iatrogenic inoculation, and contiguous spread from adjacent structures or trauma [1, 5]. Streptococci, staphylococci, *Haemophilus influenzae*, and *Neisseria gonorrhoeae* are the most common etiologies [1, 2, 6]. Suggested therapies include antibiotics, needle aspiration, arthrotomy, and arthroscopy with lavage [3].

Lemierre’s syndrome is a rare complication of oropharyngeal and otological infections and refers to an infectious thrombophlebitis of the internal jugular vein (IJV), which may be further complicated by septic embolization [7].

We present a case of septic arthritis in a 72-year-old patient with a complicated course of disease including septic shock, atrial fibrillation, cerebral abscess, Lemierre’s syndrome, and pathological fracture of the mandibular condyle. In addition, the patient had an incidental finding of intracardiac myxoma.

To our knowledge, no cases of septic arthritis of the TMJ preceding Lemierre’s syndrome have been published. In addition, only a few cases of septic arthritis of the TMJ resulting in severe systemic infection and cerebral abscess formation are reported.

## Case report

A 72-year-old male was referred to the Maxillofacial Surgery Department by his dentist because of pain and swelling of the right temporomandibular region. His medical history included hypertension and coronary heart disease. Four months earlier, he underwent elective stenting of his right coronary artery, followed by double anti-platelet therapy with clopidogrel and acetylsalicylic acid. He exercised regularly, did not smoke, and had no previous history of temporomandibular disease or immunodeficiency.

The patient reported sudden pain and a sensation of subluxation in his right temporomandibular region while yawning 3 weeks prior to hospital admission. This was followed by a gradual increase of swelling and tenderness in the TMJ region.

**Fig. 1:**
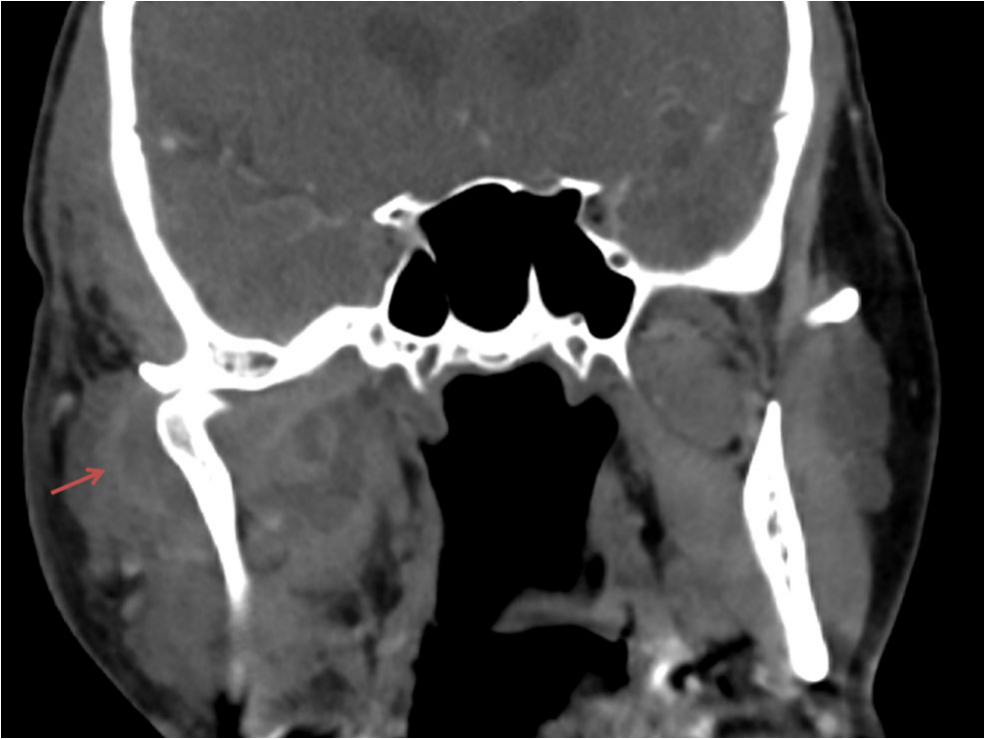
Coronal view of CT angiography of the face showing peri-articular effusion (arrow) of the right TMJ

Upon examination, the patient was hypotensive with a blood pressure of 88/65 mmHg, the heart rate was irregular with a frequency of 90–120 per minute, and he had tachypnea with a respiratory rate of 31 per minute and temperature 37.7 °C. His skin was warm and sweaty, and he appeared disoriented and responded with latency. Swelling in the right pre-auricular area that extended to the angle of the mandible was noted. Pitting edema in the temporal region was also present as well as trismus, with a maximal mouth opening of 2 cm. There was no apparent fluctuation in the skin or mucosa. A CT angiography of the face and neck showed extensive temporomandibular joint effusion along with edema in the masseter, temporal, and lateral pterygoid muscles with signs of abscess formation (Fig. 1). In addition, thrombophlebitis in the ipsilateral internal jugular vein (IJV) was seen.

Blood samples showed increased inflammatory markers with leucocytes at 18.8 cells per μL (3.5–10.0), sedimentation rate 58 mm (1–12), and C-reactive protein of 251 mg/L (< 4). Platelet count was low at 98 cells per μL (145–390), and creatinine was elevated at 172 μmol/L (60–105).

**Fig. 2:**
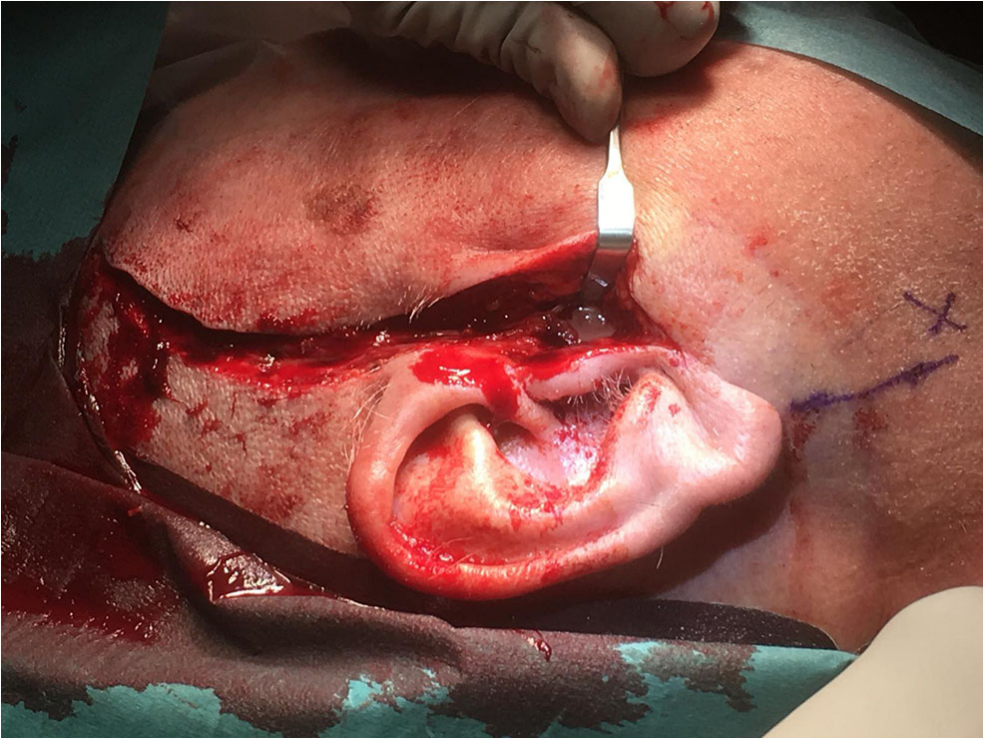
Intra-operative picture of the right TMJ which shows pus from the incised capsule

**Fig. 3:**
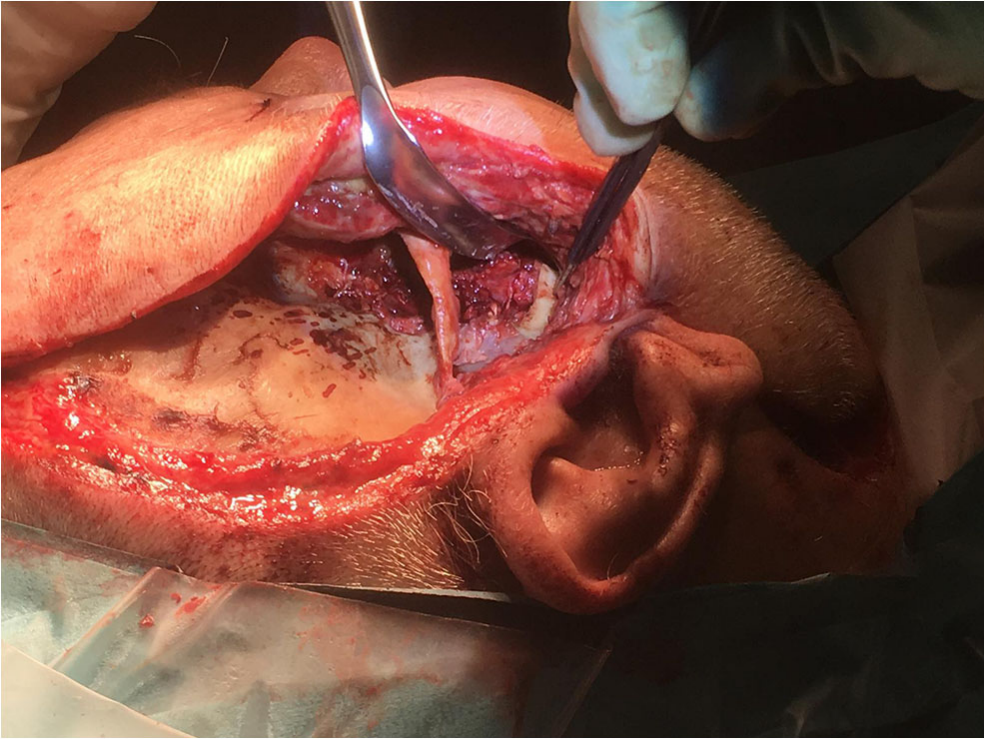
Intra-operative picture of the right temporal fossa where the temporal muscle has been removed due to necrosis

Aspiration from the temporomandibular joint revealed blood-tinged pus. A diagnosis of septic arthritis of the TMJ with peri-articular extension was made. Moreover, he had septic shock with a Sequential Organ Failure Assessment score (SOFA-score) of 12. Intravenous crystalloid fluid, norepinephrine, ceftriaxone, and metronidazole were administered, and the patient was taken directly to the operating theater. A pre-auricular incision was made with extension temporally making a hemi-coronal incision. Pus was evacuated from the temporomandibular joint, which was explored and debrided thoroughly with removal of necrotic tissue (Fig. 2). The articular disc appeared grayish in color and was removed along with the joint capsule. Because of the extensive necrotic peri-articular tissue, an intraoral vestibular incision was made with further exploration of the masseteric and pterygomandibular space. No further necrotic tissue was found on the medial or lateral side of the ramus of the mandible. Intra-operative bacterial samples with direct gram staining showed gram-positive cocci and gram-negative rods. No change in antibiotic treatment was made. Low molecular weight heparin was administered postoperatively. Further revision was made the following day where the temporal muscle was now found to be avascular and therefore removed along with necrotic tissue in the masseter and lateral pterygoid muscle (Fig. 3).

The patient required monitoring in the intensive care unit (ICU) for 11 days and underwent a total of six surgical revisions due to progression of the necrosis. Debridement of necrotic tissue in the pterygopalatine fossa, infratemporal fossa, the masseter muscle, and the pterygomandibular space was done. During the revisions, necrotic tissue was removed until viable tissue was encountered. The temporal muscle was removed in total due to development of necrosis. The coronoid process was removed to facilitate surgical access. During the ICU admission, the patient required norepinephrine to maintain adequate blood pressure in the first 8 days. Intercurrent atrial fibrillation was successfully treated with electrocardioversion at day 5. A tracheotomy was performed the same day.

*Streptococcus constellatus* was isolated from the TMJ aspirate and peri-auricular pus, sensitive to the prescribed antibiotics. All (four) blood cultures were positive for *Streptococcus constellatus*, and anaerobic blood culture was positive for *Dialister pneumosintes* prior to initiation of antibiotic treatment. Due to persistent fever despite adequate surgical and medical treatment, transthoracic and transesophageal echocardiogram was performed to rule out infectious endocarditis as the underlying etiology. This showed an incidental finding of a tumor adherent to the left atrial septum, measuring 2.3 × 2.5 cm, but no signs of endocarditis. The thrombophlebitis in the superior part of right IJV was also visualized, which extended 5 cm inferior to the jugular foramen. A tentative diagnosis of non-infectious myxoma of the heart was made. The valvular apparatus appeared normal without signs of endocarditis.

**Fig. 4:**
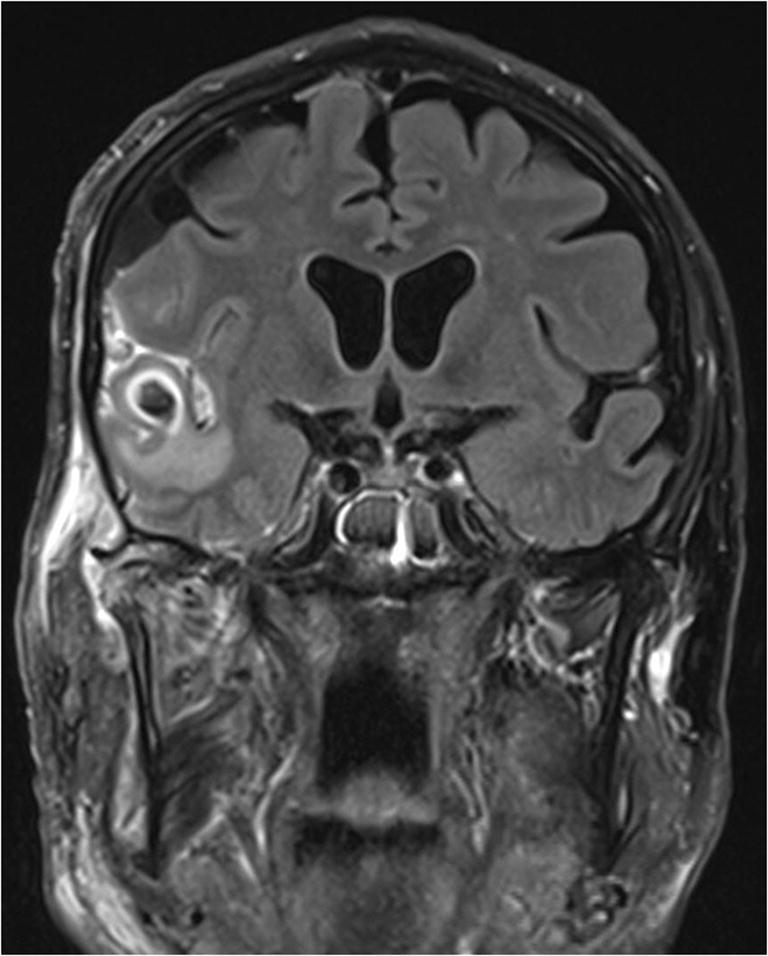
Cerebral MRI showing a contrast enhanced abscess in the right temporal lobe, measuring 1.2 × 1.3 cm

A cardiothoracic surgeon was consulted, and removal of the tumor was planned as soon as the patient recovered from the infection. A further thoracic CT was done, which in addition to the tumor in the left atrium showed signs of multiple septic emboli in both lungs. Cerebral MRI showed a cerebral abscess in the right temporal lobe, measuring 1.2 × 1.3 cm (Fig. 4), as well as a narrow empyema anterior to the temporal pole. Because of the small size, the cerebral abscess was treated non-surgically. The infectious diseases specialist recommended 6-week intravenous high-dose ceftriaxone and metronidazole, considering penetration through the blood-brain barrier and into the abscess fluid. This was followed by 3-week oral amoxicillin treatment preceding MRI control and new evaluation of treatment length.

The patient gradually improved and the tracheostomy decannulation was performed on day 11. He was awake with intact cognitive functions. He had no neurological deficits. Follow-up MRI showed full regression of the abscess, and thus antibiotic treatment was discontinued.

The patient was transferred to the cardiothoracic department on day 24 after admission. He had persistent malocclusion with open bite in the left posterior segment, and only the second molar was in occlusion on the right side. Due to the multiple organ complexity, corrective treatment of the occlusion was pending further clinical stabilization. Cardiac surgery was performed on day 26 with full removal of the myxoma which appeared smooth and non-infected. The diagnosis was confirmed histologically. He was discharged to his local hospital for further convalescence 1 month after first admission.

**Fig. 5:**
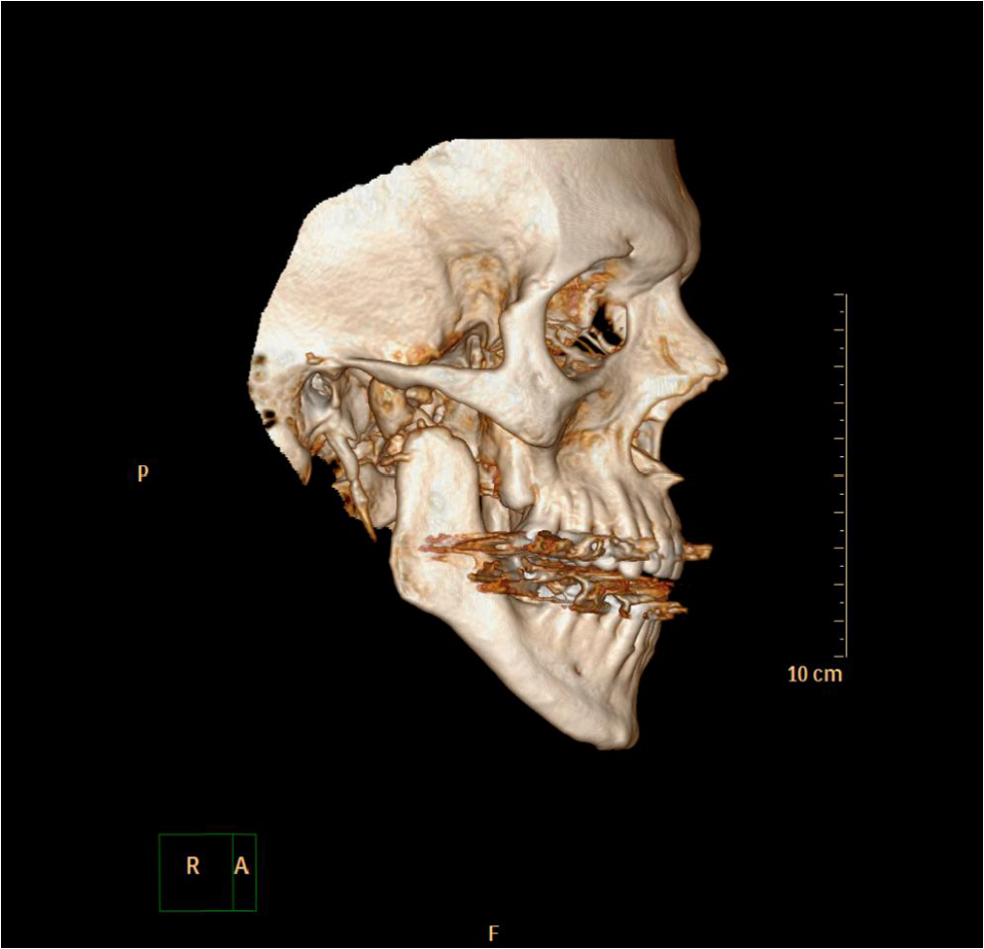
Three-dimensional reconstruction of CT which shows a pathological fracture of the right mandibular condyle

Persistent cosmetic hollowing of the right temporal region was noted at the follow-up appointments at the outpatient clinic; however, the patient rejected further surgical correction. He had reduced occlusal contact on the right posterior segment, and CT showed a pathological fracture in the right mandibular condyle (Fig. 5). Because there were no signs of persistent infection, the condylar process was not removed. He did not report any pain following hospital discharge. The occlusion was partially corrected with reduction of the cusp height on the opposite side. He had persistent reduced mouth opening but achieved an inter-incisal opening of 30 mm which was considered adequate by the patient. Owing to the absence of signs indicating persistent, chronic infection, joint replacement surgery has been suggested, but not yet scheduled.

## Discussion

Septic arthritis of the TMJ usually causes isolated locoregional symptoms such as pain, pre-auricular swelling, and malocclusion. In their study, Cai and colleagues found fewer than 40 published cases of septic arthritis of the TMJ in the English language literature. With a further 40 cases from their hospital, the total number of published cases was approximately 80 [1]. As in our patient, an initial attack of severe arthralgia was reported in 38 of their patients [1]. None of the patients in their study however had as severe disease course as our patient, which included septic shock, necrosis of peri-auricular tissues, cerebral abscess formation, and septic thrombophlebitis of the IJV [1]. Furthermore, only seven of their patients had fever, and only five had pre-auricular swelling [1]. Subtle local and systemic manifestations of septic arthritis of the TMJ may lead to a delay in diagnosis, and it may also be underreported [2]. In their case report from 2017, Xiao et al. describe a patient with a more severe infection, including extensive abscess formation requiring open surgical drainage as well as condylectomy [8]. It is, however, our impression that the majority of cases presented in the literature have a less severe course of disease.

In addition to severe locoregional findings, our patient developed an intracranial abscess. Cerebral abscesses are rare complications of maxillofacial infections, including septic arthritis of the TMJ, with streptococci being the most common reported etiology [6, 9, 10]. Streptococci were also the offending bacterial agent in our case. We believe that the cerebral abscess was caused by the infected TMJ, its surrounding tissues, or a complication of the septic thrombophlebitis of the IJV rather than septic embolization by the myxoma. Although cardiac myxomas can cause intracranial embolization, they are rarely infected [11].

Our patient did not have any of the suggested risk factors of septic arthritis of the TMJ such as upper respiratory tract infection, recent extraction of an upper molar, trauma, iatrogenic injury, or systemic or autoimmune disease [3, 4]. Nevertheless, the *Streptococcus constellatus* found in the pus aspirated from the TMJ and in blood cultures likely originated from the oral cavity. We believe that the sudden pain experienced by our patient when yawning could have been due to a tear in the joint capsule or the intra-capsular tissue. A resultant hematoma could subsequently have been infected, either by hematogenous seeding or, less likely, contiguous spread from nearby structures. Joint trauma is considered a predisposing factor for hematogenous spread [2, 8, 12]. Six of the patients in the study by Cai and colleagues had a recent history of this, either by dislocation or blunt trauma to the TMJ [1].

The subsequent development of Lemierre’s syndrome, with thrombophlebitis of the IJV and septic emboli to the lungs, is likely to have been caused by local spread of the infection from the TMJ. Lemierre’s syndrome is a rare condition usually caused by *Fusobacterium necrophorum*, but *Streptococcus constellatus* has also been reported as a possible cause [7, 13]. It primarily affects young people following an oropharyngeal infection and has a reported incidence of 3.6 cases per 1 million per year [14]. Known complications include septic emboli to the lungs, greater joints, and brain [15]. Treatment consists of i.v. antibiotic treatment and, if necessary, surgical management of the primary condition. Anticoagulation treatment is controversial [15].

To our knowledge, this is the first reported case where septic arthritis of the TMJ precipitates Lemierre’s syndrome. This is surprising, given the close anatomic proximity of the TMJ to the IJV. Thus, the combination of the two conditions may be underreported, and clinicians treating patients with septic arthritis of the TMJ should be aware of this serious complication.

In addition to the severe complication of Lemierre’s syndrome, our case illustrates that septic arthritis of the TMJ may cause serious systemic effects, such as sepsis. Thus, prompt diagnosis and treatment are necessary.
